# Encapsulated Leptin‐Producing Cells Facilitate Entrainment of Circadian Rhythms in Rodents and Nonhuman Primates

**DOI:** 10.1002/advs.77191

**Published:** 2026-08-13

**Authors:** Samantha T. Fleury, Xuanyi Lin, Peter D. Rios, Christopher Olker, Eun Joo Song, Alejandra Cobos Perez, Cody Fell, Daisy Lopez, Ira Joshi, Hafsa Nasir, Cecelia Curtis, Danna Muringi, Kaiyuan Wang, José Oberholzer, Fred W. Turek, Isaac B. Hilton, Jonathan Rivnay, Martha Hotz Vitaterna, Omid Veiseh

**Affiliations:** ^1^ Department of Bioengineering Rice University Houston Texas USA; ^2^ Center for Sleep and Circadian Biology Northwestern University Evanston Illinois USA; ^3^ Department of Neurobiology Weinberg College of Arts and Sciences Northwestern University Evanston Illinois USA; ^4^ CellTrans Inc. Chicago Illinois USA; ^5^ Department of Visceral and Transplant Surgery University of Zurich Zürich Switzerland; ^6^ Department of Biomedical Engineering McCormick School of Engineering Northwestern University Evanston Illinois USA; ^7^ Department of Materials Science and Engineering McCormick School of Engineering Northwestern University Evanston Illinois USA

**Keywords:** cell therapies, circadian alignment, leptin, metabolic regulation, shift work

## Abstract

Both the timing and composition of food intake have gained recognition for their effects on circadian (daily) rhythms in mammals, however the mechanisms by which these effects are exerted are incompletely understood. The present studies focus on synchronization of circadian rhythms to the environmental light‐dark cycle, particularly re‐entrainment following a phase shift such as occurs with travel across time zones or shift work. To examine the role and therapeutic potential of the peptide hormone leptin in these effects, we create and characterize injectable leptin factories consisting of encapsulated human retinal pigment epithelium (RPE) cells engineered to constitutively produce leptin. Subcutaneous injections of these leptin factories result in sustained elevation of blood leptin over more than a day, and accelerate entrainment to both phase delays and phase advances. Leptin factory administration to diurnal cynomolgus macaques is safe and effectively reduced entrainment rate by approximately 1 day. In addition, capsule administration in cynomolgus macaques has no demonstrable adverse effects on sleep, increasing Non‐Rapid Eye Movement sleep (NREM sleep) slow‐wave energy. Taken together, these results implicate metabolic regulation as a novel approach to accelerating adaptation to changing schedules and suggest that leptin can be used to therapeutically decrease entrainment time.

## Graphical abstract

A clinically translatable cell line can be engineered to produce leptin, encapsulated in biomaterial and safely implanted to produce and deliver the metabolic regulating protein within the body. Implantation of these encapsulated cells decreases the time it takes for mice and non‐human primates to adjust to circadian disruptions similar to those experienced during jet lag or shift work.

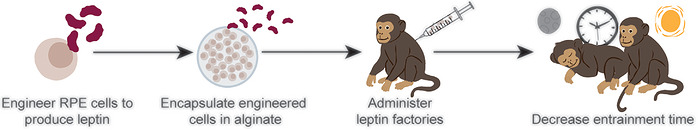

## 1 Introduction

Circadian (daily) rhythms are endogenous, near 24‐h cycles in biological processes generated in cells by a transcription‐translation feedback loop of core clock genes. In mammals, the circadian system is comprised of multiple tissue clocks, entrained by time cues, or Zeitgebers (from German, Time‐givers), such as the light‐dark cycle or behavioral cycles of sleep and wake. When stable entrainment to the environment occurs, there is alignment of rhythms across tissues and organs. However, entrainment instability can arise from challenges such as travel across time zones, shift work, or deficient or mistimed sleep. Diet composition, including fat, protein, fiber, or vitamin content, can modify circadian rhythms and sleep, while metabolic factors such as obesity have also been associated with sleep disruptions and circadian changes [1, 2, 3, 4, 5, 6, 7, 8]. Evidence suggests circadian disruption can have adverse effects on mental and physical health not only as a symptom but as a cause of cardiometabolic and neurodegenerative diseases [9]. To address these health issues, there is a critical need for approaches to facilitate adaptation to changing schedules. Controlling light exposure, changes in mealtimes and melatonin use have all been shown to decrease entrainment time [10, 11, 12]. However, these strategies all require consistent specifically timed behavior and interventions that are inconvenient or incompatible with many people's lifestyles.

Metabolism and circadian rhythms are closely linked. Feeding time can act as a Zeitgeber [13], affecting alignment of clocks [14]. Feeding time more strongly entrains peripheral tissue clocks than the central clock of the hypothalamic suprachiasmatic nuclei (SCN) which respond more strongly to light [15, 16]. The metabolic impact of high‐fat diet is compounded by feeding time with, “wrong” time feeding leading to increased weight gain and “right” time feeding mitigating the effects of high‐fat diet [17]. Both the time and the composition of food consumed can enhance or disrupt the coordination of circadian rhythms across tissues, suggesting metabolic signals may be a target for therapeutically enhancing entrainment. Metabolic hormones such as leptin, adiponectin and insulin are linked to both food intake and circadian fluctuation and may additionally act as peripheral zeitgebers [18]. The peptide hormone leptin, rhythmically produced by adipose tissue, is a candidate food‐related signal influencing entrainment. Leptin injection increases the magnitude of phase shifts to light [19, 20, 21]. However, injection of leptin fails to produce significant phase shifts by itself [19].

Engineered cell therapies are an emerging class of therapeutics that can be used produce and deliver bioactive proteins in situ. As a living therapeutic these cells can continually produce protein, alleviating some of the practical dosing challenges of delivering short lived proteins [22]. Biomaterial encapsulation such as alginate can be used to isolate the engineered cells from the immune system, allowing for the use of allogeneic cells and an off the shelf style therapy [23]. This combined strategy of biomaterial encapsulation and cell produced biologic is already approved for use clinically and continues to be explored as a way to treat a wide array of diseases [22, 24, 25].

The goal of the present studies was to explore leptin's potential role as a metabolic to circadian signal that may be used to enhance circadian entrainment. Leptin, like other peptide hormones, has multiple practical limitations, including its rapid degradation and difficulty in administration [26]. Synthetic peptides that may overcome these limitations risk immunogenicity. To do this, we developed a novel, “leptin factory” consisting of encapsulated genetically engineered cells capable of producing native leptin over an extended time period independently of feeding time or diet composition. Using these leptin factories, we were able to decrease entrainment time in mice and found that the effect translates to diurnal nonhuman primates without any toxicity or negative impact on sleep.

## 2 Results

### 2.1 Development and Characterization of a Leptin Producing Encapsulated Cell Therapy

While there is evidence that leptin plays a role in the circadian system, it has already been shown that leptin injections alone do not induce a phase response [7]. Therefore, in order to investigate leptin as a potential therapeutic way to decrease entrainment time, we designed an encapsulated cell therapy to produce leptin in situ. We chose human retinal pigmented epithelial (RPE) cells to produce and deliver the therapeutic as they are a clinically translatable cell chassis. As human cells they produce proteins with human glycosylation patterns, decreasing the immunogenicity potential of the therapeutic proteins produced and importantly for safety, RPE cells display contact inhibition, a trait that makes them nontumorigenic [27, 28].

**FIGURE 1:**
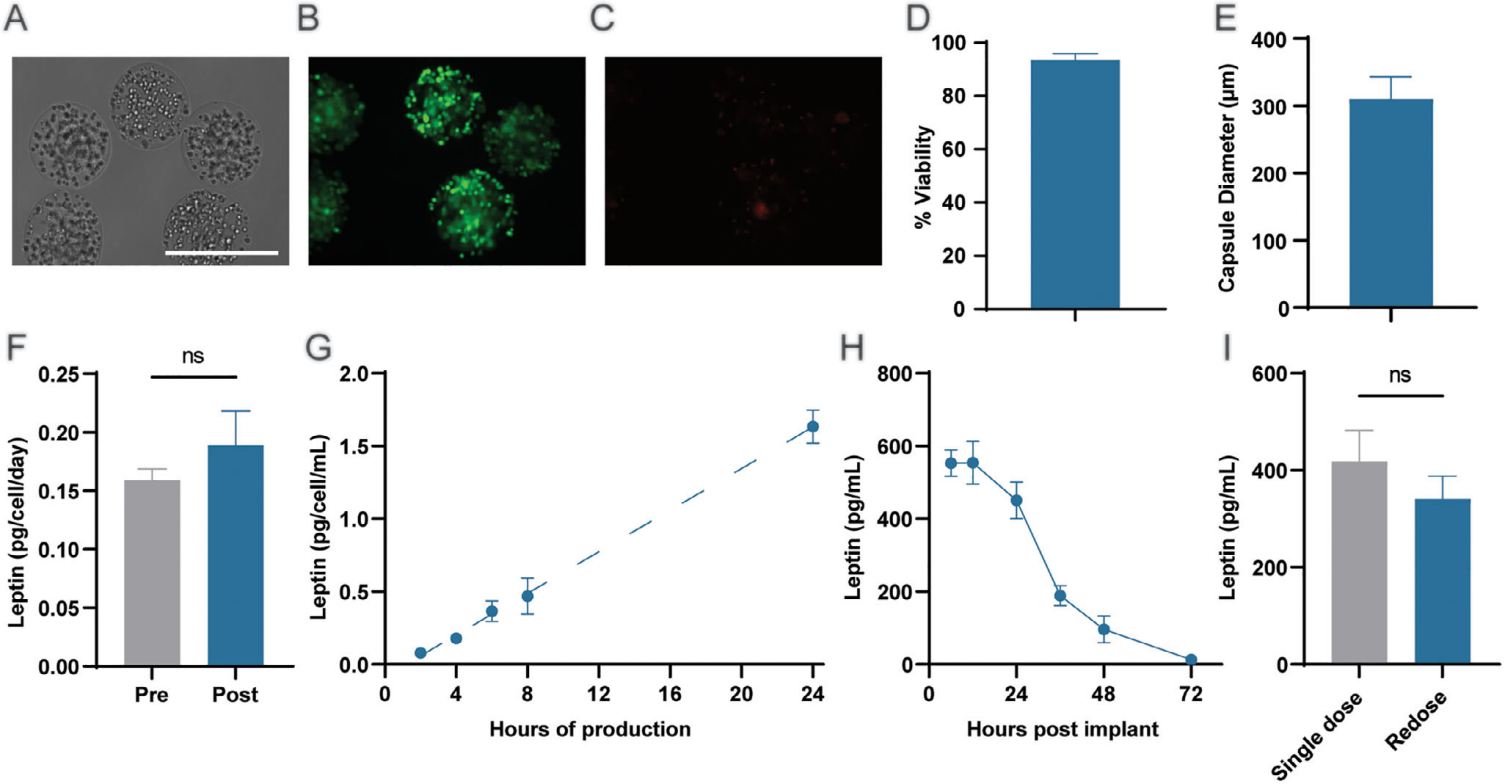
Encapsulated Engineered ARPE‐19 cells as leptin factories. (A) Representative bright field image of encapsulated ARPE‐19 cells. Scale bar = 400 um. (B) Representative image of encapsulated ARPE‐19 cells stained with calcein AM (Live) (C) and Ethidium homodimer‐1 (Dead). (D) Quantification of encapsulated cell viability by trypan blue staining after capsule lysis (N = 12). (E) Quantification of capsule size (N = 31). (F) Production of leptin from engineered ARPE‐19 cells before and after encapsulation (N = 12 pre and N = 11 post). Statistical significance calculated by unpaired t test p = 0.3343. (F) Leptin produced from encapsulated engineered ARPE‐19 cells over the course of 24 h (N = 5‐6). Dashed line is a linear regression with an R2 of 0.9143. (H) Human serum leptin concentration in mice injected with encapsulated leptin factories. (N = 6, 3M 3F). (B, I) Human serum leptin levels 24 h after mice were injected with encapsulated leptin factories for the first time (single dose) or 2 weeks after receiving a prior dose (Redose). (Single dose N = 6, 3M, 3F Redose N = 5 2M 3F). Statistical significance calculated with unpaired *t* test p = 0.3678.

After engineering our RPE cells to express leptin we encapsulated these RPE‐LEP cells in 300 µm SLG20 alginate capsules via electrospraying. To validate the cells remained viable through the encapsulation process, we live‐dead stained the capsules and collected fluorescent images to visually assess the encapsulation (Figure 1a–c). To confirm the high viability seen in the imaged capsules, we lysed capsules, counted the freed cells using a standard trypan blue assay and found that cells remained 93.5% ± 2.3% viable (Figure 1d). Consistency of capsule size was confirmed by analyzing images of encapsulated cells with a custom MATLAB code which found that capsules were 310 ± 32.87 µm (Figure 1e). The pores in alginate allow nutrients and small proteins to diffuse through freely but are too small for immune cells to enter [29]. We confirmed that production and secretion form our RPE‐LEP cells was not affected by quantifying production from both encapsulated and unencapsulated cells over 24 h and observed there was no difference in the quantity of leptin produced from encapsulated cells (p = 0.3343) (Figure 1f). Accumulation of encapsulated cell produced leptin in media was linear (R^2^ = 0.9143) (Figure 1g) indicating consistent production from our encapsulated cell therapy over 24 h.

The half‐life of native leptin in a mouse is 40.2 min [14]. To evaluate the pharmacokinetics of our encapsulated RPE‐LEP cells we injected capsules subcutaneously and monitored levels of the cell produced human leptin in the blood. Leptin was detectable in the blood for 48 h, well beyond when recombinant leptin would be degraded (Figure 1h). Given the potential need for multiple rounds of treatment, we also evaluated the effectiveness of redosing. Mice which received a dose of capsules two weeks prior were injected with a second dose of RPE‐LEP capsules alongside a group of naïve mice. We found no difference in the concentration of human blood leptin 24 h later (p = 0.3678) (Figure 1i) demonstrating RPE‐LEP capsules can be successfully dosed repeatedly.

### 2.2 Encapsulated Leptin Factories Decrease Entrainment Time

**FIGURE 2:**
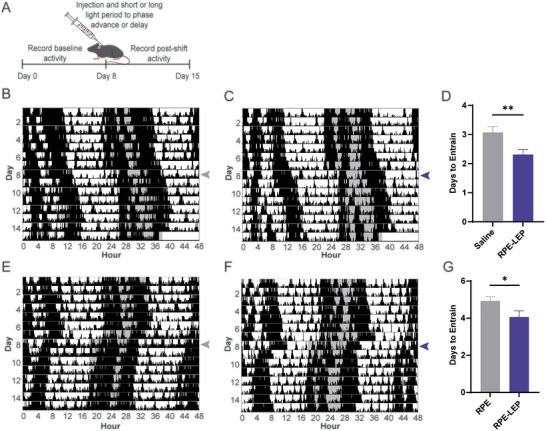
Encapsulated leptin factories decrease entrainment time in mice. (A) Overview of experimental timeline. Grey shading indicates periods of darkness. Arrows indicate the day when capsules or saline were administered. (B) Representative actogram of a mouse injected with saline or (C) encapsulated leptin factories (RPE‐LEP) and subject to a 4‐h phase delay. (D) Quantification of the number of days for mice to entrain to the new light schedule (Saline N = 15, 7M, 8F, RPE‐LEP N = 16, 8M, 8F). Statistical significance calculated with unpaired t test p = 0.0058. (E) Representative actogram of a mouse injected with unengineered ARPE‐19 containing capsules (RPE) or (F) encapsulated leptin factories (RPE‐LEP) and subject to a 4‐h phase advance. (G) Quantification of the number of days for mice to entrain to the new light schedule.

To test the efficacy of our platform we used a murine entrainment model where mice were kept on a consistent 12‐h light dark schedule leading up to a 4‐h delay or 4‐h advance in the light schedule. On the day of the change in light schedule mice were injected subcutaneously with either RPE‐LEP capsules or received a control injection. We continued monitoring mouse activity for 7 days to ensure sufficient time to entrain to the new light schedule. Entrainment time was then scored and calculated manually as described for the mice on a high fat diet. Representative actograms showing activity and light dark schedule are presented in Figure 2b,c for mice subject to a phase delay and Figure 2e,f for mice subject to a phase advance. Mice injected with saline entrained to a 4‐h phase delay after 3.07 ± 0.182 days while mice injected with RPE‐LEP capsules entrained to the same delay significantly faster, in just 2.31 ± 0.176 days (p = 0.0058) (Figure 2d).

### 2.3 Pharmacokinetics and Safety of RPE‐LEP Capsules in Non‐Human Primates

The RPE‐LEP capsules were designed with clinical translation in mind. A requirement for clinical translation is safety and efficacy in large animal models. To evaluate the translatability of our RPE‐LEP capsules we utilized cynomolgus macaques due to their diurnal rhythm and similar sleep wake patterns to humans [16].

**FIGURE 3:**
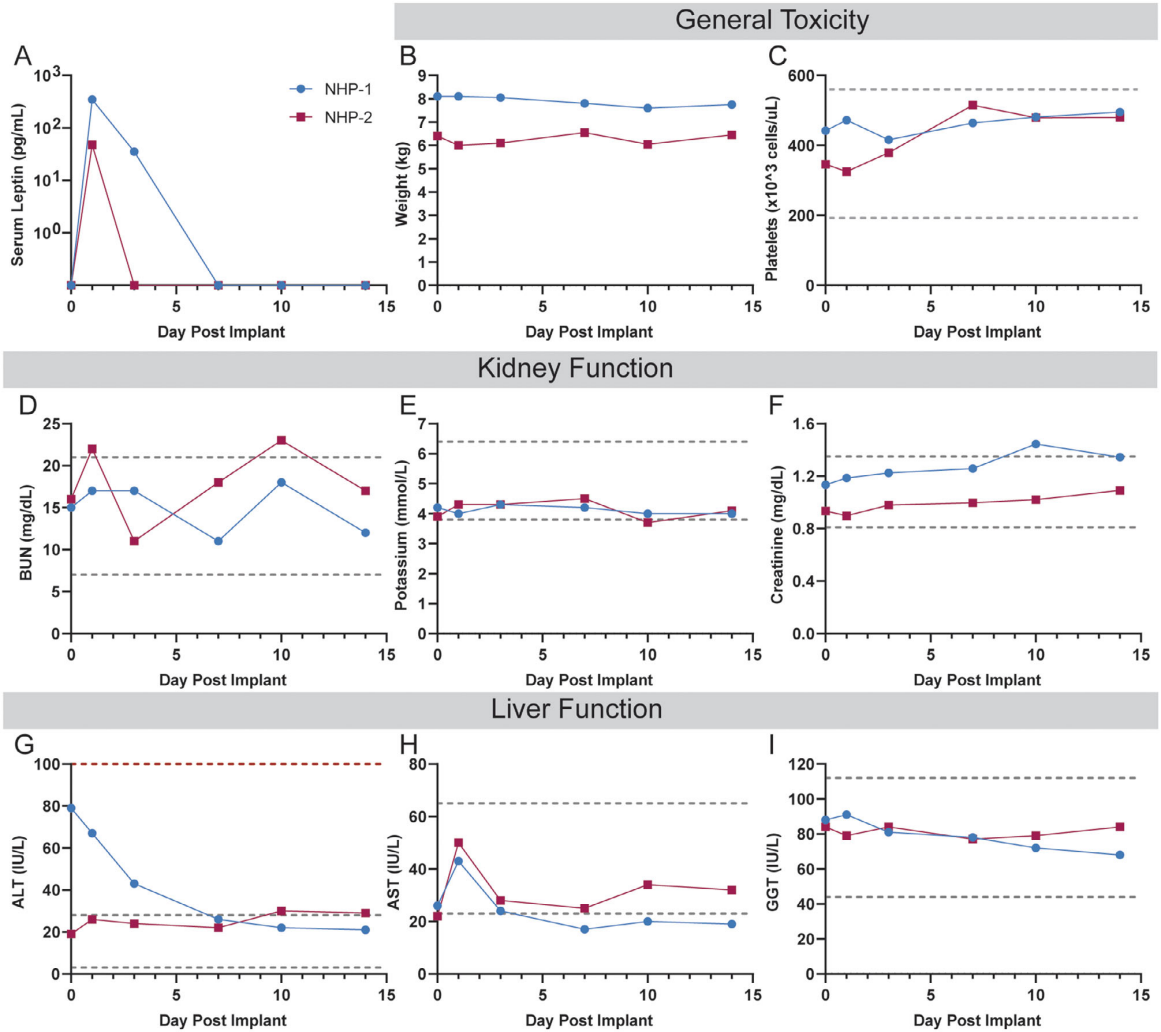
Pharmacokinetics and dosing safety of encapsulated leptin factories in non‐human primates. Grey dashed lines indicate normal ranges. Red dashed lines indicate level of clinical concern. (A) Levels of human leptin in NHP serum after injection with encapsulated leptin factories. (B) Weight of NHPs following injection with encapsulated leptin factories. (C) Platelet counts following injection with encapsulated leptin factories. (D) Blood urea Nitrogen (BUN) (E) Potassium and (F) Creatinine levels of NHPs as measures of kidney function following injection of encapsulated leptin factories. (G) Alanine aminotransferase (ALT) (H) aspartate aminotransferase and (I) gamma‐glutamyl transferase (GGT) levels as measures of liver function following injection with encapsulated leptin factories.

To assess the pharmacokinetics and safety of RPE‐LEP capsules we injected capsules subcutaneously into two animals. Periodic blood draws were taken for 14 days and a complete blood count and blood chemistries were performed along with an ELISA for the human leptin produced from the implanted capsules. Leptin showed similar pharmacokinetics in NHPs to that seen in the mice, with an increase in human leptin levels in the serum that became undetectable by days 3–7 (Figure 3a). Following injection, NHPs showed no signs of toxicity. Body weight and platelet counts remained normal, as did kidney function assessed by blood urea nitrogen (BUN), potassium and creatinine. Liver function was similarly normal as measured by alanine aminotransferase (ALT), aspartate aminotransferase (AST) and gamma‐glytamyl transferase (GGT) levels (Figure 3b–i). Any deviations from the normal range were transient, remained below the level of clinical concern and resolved without intervention suggesting that the leptin capsules were well tolerated.

Weights of the NHPs were tracked periodically over the course of a year during which the animals received multiple injections of leptin capsules. Although there were some temporary fluctuations in weight immediately after procedures there were no overall weight loss trends that would suggest toxicity or long‐term effects of the leptin (Figure S1). Histology taken from the implant site two weeks after injection shows intact capsules with localized pockets of immune cell infiltration and fibrosis near each capsule (Figure S2). Capsules retrieved two weeks after implant remain structurally intact with few viable cells remaining confirming the therapy does not persist long term (Figure S3).

### 2.4 Encapsulated Leptin Factories Decrease Entrainment Time in Non‐Human Primates Without Disrupting Sleep

**FIGURE 4:**
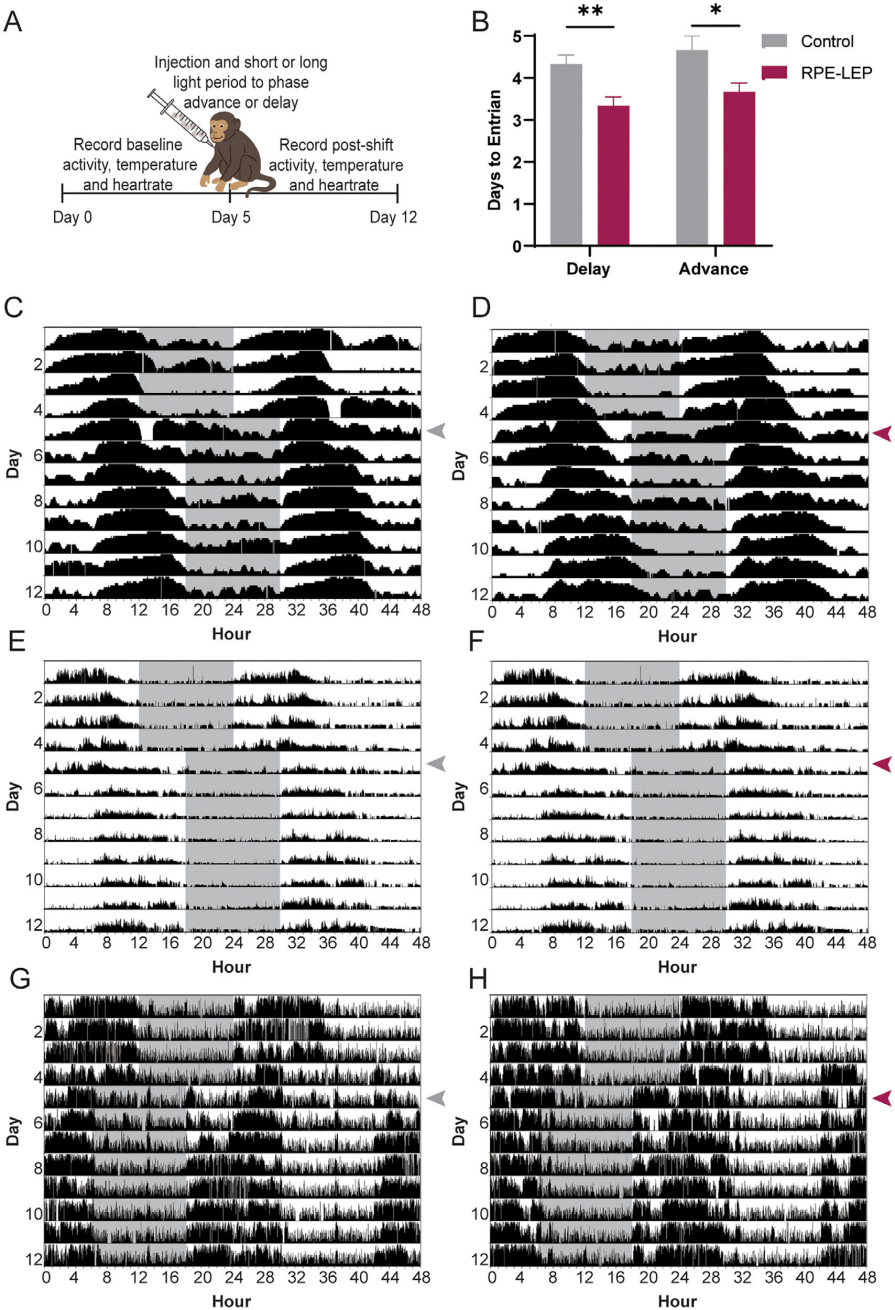
Encapsulated leptin factories decrease entrainment time in non‐human primates. (A) Overview of experimental timeline. (B) Quantification of the number of days for NHPs to entrain to the new light schedule (N = 6 2 actigraphy, 2 heartrate, 2 body temperature). Statistical significance calculated with unpaired t test (delay p = 0.0073 advance p = 0.030). Grey shading indicates periods of darkness. Arrows indicate the day when capsules or saline was administered. (C) Representative actogram of a primate injected with saline or (D) encapsulated leptin factories (RPE‐LEP) and subject to a 6‐h phase delay. (E) Representative heartrate of a primate injected with saline or (F) encapsulated leptin factories (RPE‐LEP) and subject to a 6‐h phase delay. (G) Representative body temperature of a primate injected with saline or (H) encapsulated leptin factories (RPE‐LEP) and subject to a 6‐h phase advance.

In order to validate if the decreased entrainment time we saw in mice was likely to translate to humans, we injected RPE‐LEP capsules into cynomolgus macaques and then subjected them to a 6‐h phase delay or phase advance. The non‐human primates were implanted with DSI PhysioTel telemetry implants that tracked core body temperature, heartrate and activity. As with the mice, body temperature (Figure 4c,d), heartrate (Figure 4e,f) and actigraphy (Figure 4g,h) were scored based on the number of days needed to align with the new light cycle following each shift. We then pooled all the rhythms to determine entrainment time and found that non‐human primates injected with RPE‐LEP capsules entrained to both phase delays (p = 0.0073) and phase advances (p = 0.030) significantly faster than control injected animals (Figure 4b).

**FIGURE 5:**
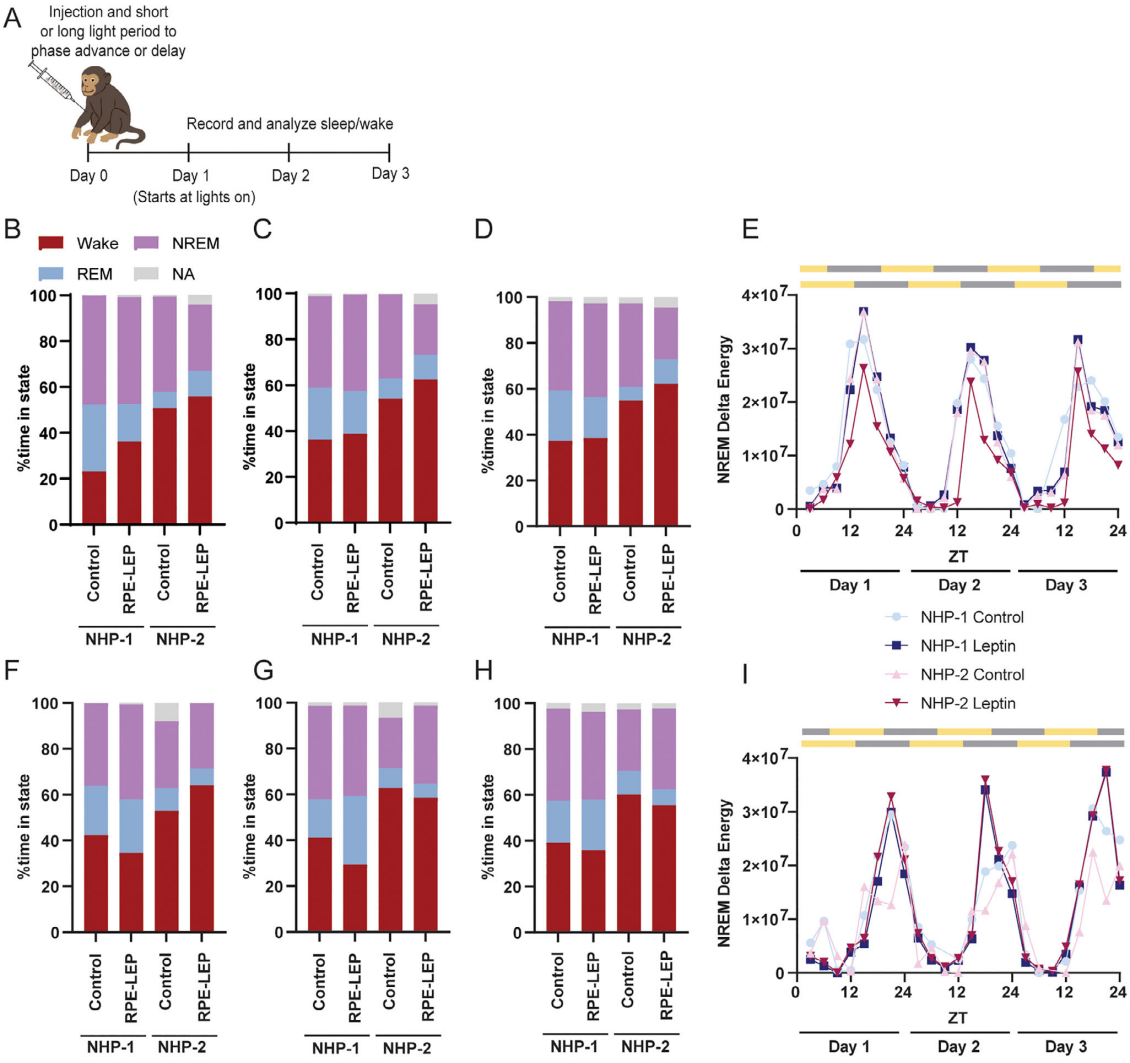
Encapsulated leptin factories do not negatively affect sleep in non‐human primates. (A) Overview of experimental timeline. (B) Time spent in Wake, NREM, REM on day 1 (C) day 2 and (D) day 3 after capsule injection and phase delay (E) NREM Delta energy for 3 h periods for the three days following capsule injection and phase delay. The yellow and grey bar represents the light dark cycle before (top) and after (bottom) the shift. (F) Time spent in Wake, NREM, REM on day 1 (G) day 2 and (H) day 3 after capsule injection and phase advance (I) NREM Delta energy for 3 h periods for the three days following capsule injection and phase advance. The yellow and grey bar represents the light dark cycle before (top) and after (bottom) the shift.

To further investigate the effect of our leptin producing capsules, we analyzed the primates’ sleep for the three days following capsule administration. Time spent awake, in rapid eye movement (REM) and non‐REM (NREM) sleep were classified by EEG and EMG readings analyzed by a combination of an experienced polysomnographer with a multiple machine‐learning classifier system. There was no consistent difference in the time spent in each sleep state in the three days following capsule administration when the primates received leptin producing capsules or a control injection. This lack of difference was true for both animals subject to a phase delay (Figure 5b–d) or phase advance (Figure 5f–h). We then examined sleep quality as measured by NREM delta energy. Following capsule administration and phase delay there was no consistent difference in NREM delta energy between groups receiving control and treatment injections (Figure 5e). However, on nights two and three following a phase advance, the primates had increased NREM delta energy during the first half of the night after receiving leptin capsules (Figure 5i). This increase in slow wave sleep suggests leptin capsule administration increases sleep quality in the nights following a phase advance.

## 3 Discussion

Here we find that the metabolic hormone leptin interacts with the circadian system and decreases entrainment time. Through the development of an encapsulated leptin producing cell therapy, we were able to create a translatable therapeutic that decreases entrainment time in both mice and nonhuman primates.

Using a clinically translatable RPE cell line, we engineered cells to stably produce leptin and then encapsulated them within alginate. The small pore size of the alginate allows for diffusion of therapeutic proteins and nutrients while preventing direct interaction of immune cells and the encapsulated RPEs [29]. The 300 µm capsules were easily administered via subcutaneous injection and designed to encourage fibrosis of the biomaterial [17] in order to create a short term therapy. Capsules explanted after 14 days contained few viable cells and fibrotic growth and immune cells were present around the capsules demonstrating that the immune response possibly in combination with the hypoxic environment around subcutaneous implants was sufficient to prevent long term viability of the therapy [30]. When injected into mice, the leptin factories temporarily elevated blood leptin levels and could be repeatedly dosed without loss of potency. We then evaluated the effect of our leptin capsules on the circadian system in mice. Mice injected with leptin capsules more rapidly entrained to both phase delays and phase advances of the light‐dark cycle. This decreased entrainment time reduces the time their circadian system is dysregulated—a therapeutically beneficial effect, as factors that cause long term disruptions in circadian amplitude are associated with increased risk of pathologies including obesity, type 2 diabetes, cardiometabolic disease and depression [8, 16].

Circadian rhythm research in nocturnal rodent models does not always translate to humans, thus we utilized cynomolgus macaques who have a diurnal rhythm and similar sleep wake patterns to humans to assess the translatability of our therapy [16]. Additionally the use of a larger animal model allowed us to look at multiple physiological rhythms in addition to the behavioral activity rhythm. We confirmed that subcutaneous injection of leptin capsules led to a similar, days‐long increase in leptin levels to that observed in mice and additionally discovered our therapy was well tolerated in non‐human primates. After 14 days the cells within the capsules are no longer viable and animals had no sustained weight loss over the course of a year. Previous studies implanting similar alginate capsules producing IL‐2 demonstrate safety out to 120 days in non‐human primates and progressed to human trials [31]. However, before translation, further studies should include long term safety outcomes and investigation of the systemic immune response to the therapeutic.

The decreased entrainment time seen in response to leptin capsule injections was preserved in non‐human primates, indicating a possible conservation of mechanism between the nocturnal mice and diurnal non‐human primates. When we evaluated sleep states in the days following capsule injection and phase shifts, we concluded there was no difference in time spent awake, in REM sleep or in NREM sleep. However, when we looked specifically at NREM delta energy, a measure of restorative slow wave sleep, we found an increase in NREM delta energy in the days following phase advance when the non‐human primates received leptin capsules. This increase suggests that although the capsules do not increase the duration of sleep, they do improve the sleep quality following a phase advance. This finding is also supported by previous research which found that leptin administration affects NREM sleep [19]. Taken together, safety and efficacy results in non‐human primates demonstrate the potential for leptin administration to be used therapeutically to decrease entrainment time.

Connections between metabolism and circadian systems are well established [32]. Mice on a high‐fat diet have an lengthened circadian period, reduced amplitude of core clock gene expression, and changes in circulating levels of metabolic hormones [4]. Previous work has demonstrated that leptin plays a role in the regulation of the circadian system. Ob/Ob mice, which lack functional leptin entrain slower to phase advances and have larger phase shifts in response to light pulses, a phenotype which is recovered by injecting leptin [8]. However, leptin is not a zeitgeber as is the case with more canonical circadian regulators such as light or melatonin, and single leptin injections do not cause a phase shift response [7]. It may be that leptin's signaling metabolic state among clocks in different tissues, modulates the responsiveness to zeitgebers rather than acting as a time cue per se, making it valuable in combination with other circadian interventions.

The increase in blood leptin concentration from leptin factories is more than an order of magnitude lower than circulating blood leptin levels in healthy adults [33]. Further investigation is needed, however the effectiveness of this modest increase may be due to the duration of leptin elevation extending beyond the typical circadian oscillations of blood leptin levels and the overall area under the curve of leptin exposure [4]. Others have demonstrated that an acute elevation of leptin by injection just prior to a 30‐minute light pulse can enhance the phase‐shifting effect of that light pulse [19], while by contrast, our cell factories produce a modest, but significantly longer‐lasting elevation of leptin in the context of exposure to a full 12‐h photoperiod. The phase‐shifting response to light is increased by the duration of light exposure [34, 35] and it is possible that a small, leptin‐induced increase in responsiveness to light over a long duration of light exposure could result in more rapid entrainment.

Leptin factories facilitated the entrainment of both physiological and behavioral rhythms in non‐human primates. However, the mechanism(s) by which leptin speeds entrainment remain unclear; leptin may affect the circadian system indirectly and in multiple ways. Leptin may be influencing behavioral changes such as food intake or impacting other metabolic factors that then influence circadian rhythms. Both mechanisms are plausible: the expression of circadian behaviors can have powerful effects on molecular rhythms [36] and metabolic status can act on circadian rhythms as well [37]. Further work to discern the contributions of these and other mechanisms to leptin's entrainment‐enhancing effects is needed for leptin to be effectively used as a circadian therapeutic. Our studies suggest the potential for leptin as a tool in enhancing entrainment, but do not elucidate mechanism.

Although the administration method is more invasive than oral melatonin or behavioral changes, administration of leptin producing cells offers practical advantages as a method for decreasing entrainment time. Unlike the light pulses, timed activity and meals or melatonin dosing currently used to accelerate entrainment [20, 21], administration of leptin factories does not require precise timing and causes a greater magnitude of shift than light alone or melatonin administration [38] An implant could also facilitate repeated re‐entrainment if multiple shifts happen in a short period of time. Additionally, cell‐produced leptin effects are temporary and reversible, making this an attractive approach for enhancing entrainment while avoiding adverse effects of chronic circadian dysregulation.

This research provides further evidence for the interplay of metabolism and circadian system and offers a proof‐of‐concept technology to use this interaction therapeutically. However, additional research is needed to better understand the nature of this metabolic‐circadian interaction and the specific mechanism of action responsible for leptin's effect. The encapsulated cell technology developed in this study is a platform technology that can be used in future research on leptin and on the effect, mechanism and therapeutic potential of other metabolic proteins. Translation of the technology as a therapeutic would benefit from refinement of the encapsulated cell technology, including using tunable and repeatedly activatable systems to improve entrainment to different magnitudes of or repeated schedule shifts.

The encapsuled cell therapy designed in the study could be adapted to therapeutically modulate the circadian system and enhance entrainment. Modification of the cell capsules could improve their sleep or circadian benefits. Efficacy of these capsules in decreasing entrainment time translated to non‐human path forward for clinical translation to regulate circadian rhythms.

## 4 Conclusion

Metabolic regulation is one mechanism through which the circadian system can be regulated. We demonstrated this by creating a translatable encapsulated engineered cell therapy that produces and delivers leptin in situ. In both rodents and non‐human primates administration of this therapy leads to decrease in entrainment time to both phase advances and phase delays without negatively impacting sleep quality. This encapsulated cell therapy is a tool that can be used to further insight into the interplay of the metabolism and circadian rhythms and offers a potential therapeutic avenue through which to treat patients experiencing circadian dysregulation.

## 5 Experimental Section/Methods

### 5.1 Cell Culture and Engineering

Human ARPE‐19 cells were obtained commercially from American Type Culture Collection (ATCC). Cells were cultured complete media made from Dulbecco's modified Eagle's medium (DMEM/F12) (Cytiva) with 10% fetal bovine Serum (FBS) (Cytiva) and 1% antibiotic‐antimitotic (AA) (Gibco).

ARPE‐19 cells were stably engineered using a piggybac transposon system transfected into the cells using Lipofectamine 3000 (Gibco). For transfection, ARPE‐19 cells were seeded into six well plates at a density of 3e5 cells/well and allowed to adhere overnight at 37°C in a 5% CO2 humidified atmosphere. Cells were then transfected according to the manufacturer's protocol with a 1:2 ratio of helper plasmid to expression plasmid. Eight hours after transfection media was replaced with fresh complete media. 24 h after transfection media was replaced with complete media containing 2 µg/mL of Puromycin (Gibco). Cells were kept under puromycin selection for 2 weeks with media replacement every 48 h before being assayed for productivity.

### 5.2 Cell Encapsulation

Prior to encapsulation cells were lifted using 0.25% trypsin and washed two times with calcium free Krebs solution. Following the second wash cells were pelleted by centrifuging at 500g for 5 min and resuspended in sterile alginate (1.4% (w/v) SLG20 Pronova dissolved in 0.8% saline) at a concentration of 2e7 cells/mL. Encapsulation was done via electrospraying using a custom built device consisting of a syringe pump (Harvad Apparatus) extruding alginate from a 30g blunt tipped needle with a voltage generator (Gamma High Voltage) attached to the needle and grounded to a glass dish containing barium crosslinking solution (1:4 barium chloride:mannitol) placed about 5 mm below the tip of the needle. The size of the capsules was adjusted by changing the voltage, with a voltage of about 3.4 kV maintaining capsules 300 um in diameter. After electrospraying capsules were crosslinked for 10 min before being washed 3x with Hepes buffer and transferred to cell culture media.

### 5.3 Cell Viability After Encapsulation

Viability of encapsulated cells was calculated by degrading the alginate containing the cells by incubating 10 uL of capsules at 37C for 10 min in 50 uL of a solution containing 1 mg/mL alginate lyase (Sigma) and 50 mm EDTA (Gibco) in DMEM/F12 media (Cytiva). Following the breakdown of the alginate the cells were mixed 1:1 with trypan blue and counted using a Countess 3 cell counter (Invitrogen).

### 5.4 Live Dead Imaging of Capsules

Capsules were washed with Dulbecco's phosphate buffered saline (DPBS) (Cytiva) and then incubated 2 um Calcein AM (Invitrogen) and 4 um ethidium homodimer‐1 (Invitrogen) in DPBS for 20 min at 37C. Excess stain was removed by washing with DPBS and capsules were imaged with an EVOS fluorescent microscope.

### 5.5 Cell Productivity Assay

To test pre‐encapsulation productivity 10 000 cells were plated per well of a 96 well plate and allowed to adhere overnight. To test productivity post encapsulation 10 uL of capsules were added to each well of a 96 well plate. In both assays media was then replaced with 200 uL of fresh media in cells were incubated for 24 h. Supernatant was then collected and assayed for leptin concentration using an Abcam human leptin ELISA (ab178994). The number of cells in the encapsulated samples was then counted using the same methods described in the cell viability after encapsulation section and a per cell productivity was calculated for each group.

### 5.6 Mouse Studies

All mouse experiments were approved by the Institutional Animal Care and Use Committee(s) (IACUCs) of Rice University and/or Northwestern University. All biological samples implanted into animals were approved by the Institutional Biosafety Committee at Rice University and Northwestern University.

C57BL/6 mice were used for all in vivo studies. For pharmacokinetic studies, these were obtained from Charles River, while for circadian behavior studies, animals were obtained from the Jackson laboratory.

### 5.7 Pharmacokinetic Studies

For pharmacokinetic studies all mice were injected subcutaneously with 250 uL of capsules suspended in 750 uL of sterile saline. Blood leptin levels were evaluated by collecting blood samples at the stated timepoints. Blood was then allowed to clot at room temperature for 15 min and then spun at 2000 RPM for 10 min to isolate serum. Leptin levels in the serum were assayed with the R&D human leptin ELISA (DLP00) run according to the manufacturer's protocol.

### 5.8 Activity/Rest Rhythms

Mouse activity/rest data was collected using the Signal Solutions (Lexington KY) PiezoSleep system, which noninvasively records movement and vibration at the cage floor. Mice were individually caged in standard “shoebox” cages with hardwood chip bedding, and standard chow and water were available ad libitum in the wire bar lid. Cages were maintained, 8 per cabinet, in ventilated, light‐tight cabinets with timer control of LED lighting. Activity data was analyzed using ClockLab Data Analysis software (Actimetrics, Inc., Wilmette, IL). Phase advances were achieved by shortening the period during which the mice were injected, and phase delays were achieved by elongating the period during which the mice were injected.

### 5.9 Nonhuman Primate Studies

Adult male Mauritian cynomolgus macaques were used for this study. All animal experiments and procedures were approved by the IACUC of University of Illinois at Chicago (UIC) and performed in accordance with the guidelines for Care and Use of Laboratory Animals of UIC.

### 5.10 Pharmacokinetic and Toxicity Studies

Capsule injection for nonhuman primates was performed under ketamine (10 mg/kg IM) sedation. The injection site was shaved and sterilized. 8 mL of capsules and 25 mL of sterile saline was then injected subcutaneously through a 14G catheter. Animals were anesthetized at 1, 3, 7, 10, and 14 days following injection for blood draws. Samples were then analyzed for complete blood counts (CBCs) and blood chemistries. Serum samples were isolated from the whole blood and leptin concentration was assayed with the R&D human leptin ELISA (DLP00) run according to the manufacturer's protocol.

### 5.11 Sleep and Circadian Rhythm Data

NHP data was collected using DSI PhysioTel telemetry implants (Data Sciences International, New Brighton, MN). Electroencephalogram (EEG), Electrocardiogram (ECG), body temperature, and activity (accelerometer) data were collected continuously from untethered animals using a transceiver positioned within proximity of their two cages. The individuals were housed in separate cages in a standard vivarium room throughout the study and were given their meal at the same time each day. On the day of injection, the light period was lengthened or shortened to phase delay or advance animals. Heart rate, body temperature, and activity data were imported and analyzed using ClockLab Data Analysis. The EEG data was scored as wake, Rapid Eye Movement (REM) sleep, and non‐REM (NREM) sleep. At least one hour/day was scored by an experienced polysomnographer, with a multiple machine‐learning classifier system used to extrapolate and complete the scoring (15). Machine scoring was then reviewed and adjusted by human scoring for ambiguous epochs. Day 1 post implant was defined as starting the first time the lights were turned on following implantation.

### 5.12 Circadian Rhythm Parameters

Unless specified otherwise, both mice and NHPs were maintained in a 12 h light: 12 h dark light cycle. Administration of encapsulated cells was performed as described for pharmacokinetic studies. For time points during the dark phase (mice only), this was performed using a dim red (Kodak safelamp, #2 filter) 15W light.

Phase shifts were determined using activity onset or offset as a phase reference point. Using ClockLab, separate lines were fit through the points before and after manipulation, and phase shift magnitude was measured as the time difference between the two lines on the day of manipulation (e.g., injection of cells).

Rate of entrainment was similarly determined using activity onset or offset as a phase reference point. Entrainment was scored blind to treatment, using ClockLab software to identify activity onset or other phase reference points in the rhythm, and the number of days/cycles required to achieve a phase angle relative to the new light‐dark cycle within 30 min of the phase angle observed before the phase shift of the light‐dark cycle.

## Author Contributions

S.T.F, P.D.R, J.O., F.W.T, I.B.H., J.R., M.H.V., and O.V. conceived the study. S.T.F and M.H.V wrote the paper. K.Y. designed and created the leptin expression plasmid. S.T.F., A.C.P., C.F, and C.C. encapsulated leptin cells and completed the in vitro characterization of the encapsulated cells, S.T.F and D.M. completed murine pharmacokinetic and dosing studies. C.O., E.J.S. and M.H.V. completed murine circadian studies. P.D.R., D.L., I.J., H.N., performed non‐primate work. X.L. performed analysis of non human primate sleep data. O.V., J.R., I.B.H. and M.H.V acquired funding.

## Funding

This study was supported by 711 Human Performance Wing (HPW) and Defense Advanced Research Projects Agency (DARPA) under agreement number FA8650‐21‐1‐7119.

## Conflicts of Interest

The authors declare no conflicts of interest.

## Supporting information




**Supporting File**: advs77191‐sup‐0001‐SuppMat.docx.

## Data Availability

All data needed to evaluate the conclusions in the paper are present in the paper and/or the Supplementary Materials. The data can be provided by Omid Veiseh pending scientific review and a completed material transfer agreement. Requests for any data should be submitted to: omid.veiseh@rice.edu
