# Supplementary material for: Encapsulated Leptin‐Producing Cells Facilitate Entrainment of Circadian Rhythms in Rodents and Nonhuman Primates
Source: Adv Sci (Weinh). 2026 Aug 13;13(61):e77191. doi: 10.1002/advs.77191 (PMC13472107; doi:10.1002/advs.77191)
Supplement: Supplementary file 1 [file ADVS-13-e77191-s001.docx]

**Figure S1. Long term weights of non-human primates.** Grey arrows indicate injection of leptin factories

**Figure S2. H&E staining of implant site** from A) NHP-1 B) NHP-2. Yellow boxes show location of C) from NHP-1 and D) from NHP-2

**Figure S3. Explanted Capsules** A) Brightfield B) Stained for viability
